# Regulation of the antiapoptotic protein cFLIP by the glucocorticoid Dexamethasone in ALL cells

**DOI:** 10.18632/oncotarget.24782

**Published:** 2018-03-27

**Authors:** Lara Kleinesudeik, Katharina Rohde, Simone Fulda

**Affiliations:** ^1^ Institute for Experimental Cancer Research in Pediatrics, Goethe-University, Frankfurt, Germany; ^2^ German Cancer Consortium (DKTK), Partner site Frankfurt, Germany; ^3^ German Cancer Research Center (DKFZ), Heidelberg, Germany

**Keywords:** apoptosis, cFLIP, glucocorticoid, Smac mimetic

## Abstract

We recently reported that the Smac mimetic BV6 and glucocorticoids, e.g. Dexamethasone (Dexa), synergize to induce cell death in acute lymphoblastic leukemia (ALL) *in vitro* and *in vivo*. Here, we discover that this synergism involves Dexa-stimulated downregulation of cellular FLICE-like inhibitory protein (cFLIP) in ALL cells. Dexa rapidly decreases cFLIP_L_ protein levels, which is further enhanced by addition of BV6. While attenuating the activation of non-canonical nuclear factor-kappaB (NF-κB) signaling by BV6, Dexa suppresses cFLIP_L_ protein but not mRNA levels pointing to a transcription-independent downregulation of cFLIP_L_ by Dexa. Analysis of protein degradation pathways indicates that Dexa causes cFLIP_L_ depletion independently of proteasomal, lysosomal or caspase pathways, as inhibitors of the proteasome, lysosomal enzymes or caspases all failed to protect from Dexa-mediated loss of cFLIP_L_ protein. Also, Dexa alone or in combination with BV6 does not affect overall activity of the proteasome. Importantly, overexpression of cFLIP_L_ to an extent that is no longer subject to Dexa-imposed downregulation rescues Dexa/BV6-mediated cell death. Vice versa, knockdown of cFLIP increases BV6-mediated cell death, thus mimicking the effect of Dexa. Altogether, these data demonstrate that Dexa-mediated downregulation of cFLIP_L_ protein promotes Dexa/BV6-mediated cell death, thereby providing novel insights into the synergistic antitumor activity of this combination treatment.

## INTRODUCTION

Apoptosis is one of the best characterized forms of programmed cell death and a crucial process in physiological and pathophysiological conditions [1]. Its execution is tightly regulated by several proteins, like Inhibitor of Apoptosis (IAP) proteins [2] or cFLIP [3]. Evasion of programmed cell death is a hallmark of cancer and accomplished, for example, by aberrant expression of antiapoptotic proteins.

One major cell death regulator is cFLIP. High cFLIP expression is correlated with a poor prognosis in several tumor entities [4–6] and its downregulation is part of effective drug-mediated cell death [7]. There are two main isoforms of cFLIP expressed in human cells which control cell death in a distinct manner: The long isoform cFLIP_L_, a 55 kDa protein, and the short isoform cFLIP_S_, a 25 kDa protein [3]. cFLIP_L_ is a caspase-8/-10 homolog with two death effector domains (DEDs), but with an inactive caspase domain. Its influence on cell death regulation is being controversially discussed, as it is reported to exert pro- or antiapoptotic effects, depending on the context. Lower levels of cFLIP_L_ are associated with a proapoptotic function, as highly active heterodimers are formed [8]. In higher concentrations, cFLIP_L_ prevents caspase-8 activation in the death-inducing signaling complex (DISC) [9], which is formed upon death receptor-mediated cell death and caspase-8 activation in the ripoptosome, a signaling platform formed upon IAP depletion [10, 11]. cFLIP_S_ consists only of two DEDs and directly inhibits caspase-8 activation at the DISC [9] and the ripoptosome [10, 11] and thereby apoptosis.

Other cell death-regulating proteins are IAP proteins which are known to be prognostic factors in different tumor entities [12]. Their inhibition as therapeutic approach has been intensively studied and IAP antagonists including Smac mimetics have been developed. Smac mimetics, e.g. BV6, induce autoubiquitination and proteasomal degradation of IAP proteins and have been shown to promote cell death induced by different stimuli [13].

New treatment strategies are required for ALL, as the prognosis for relapsed patients is still poor [14]. We previously discovered that the Smac mimetic BV6 sensitizes ALL cells to glucocorticoids, such as Dexa, which are part of the standard therapy of ALL patients, by promoting the formation of the ripoptosome complex and by exerting antileukemic activity in a patient-derived xenograft model of ALL *in vivo* [15]. The assembly of the ripoptosome is regulated, amongst others, by the two major isoforms of cFLIP, i.e. cFLIP_L_ and cFLIP_S_ [10, 11]. Therefore, we studied the role of cFLIP in regulating Dexa/BV6-mediated cell death to gain new insights into the molecular mechanisms underlying the synergism of Dexa and BV6.

## RESULTS

### Dexa downregulates cFLIP_L_ protein in ALL cells

**Figure 1:**
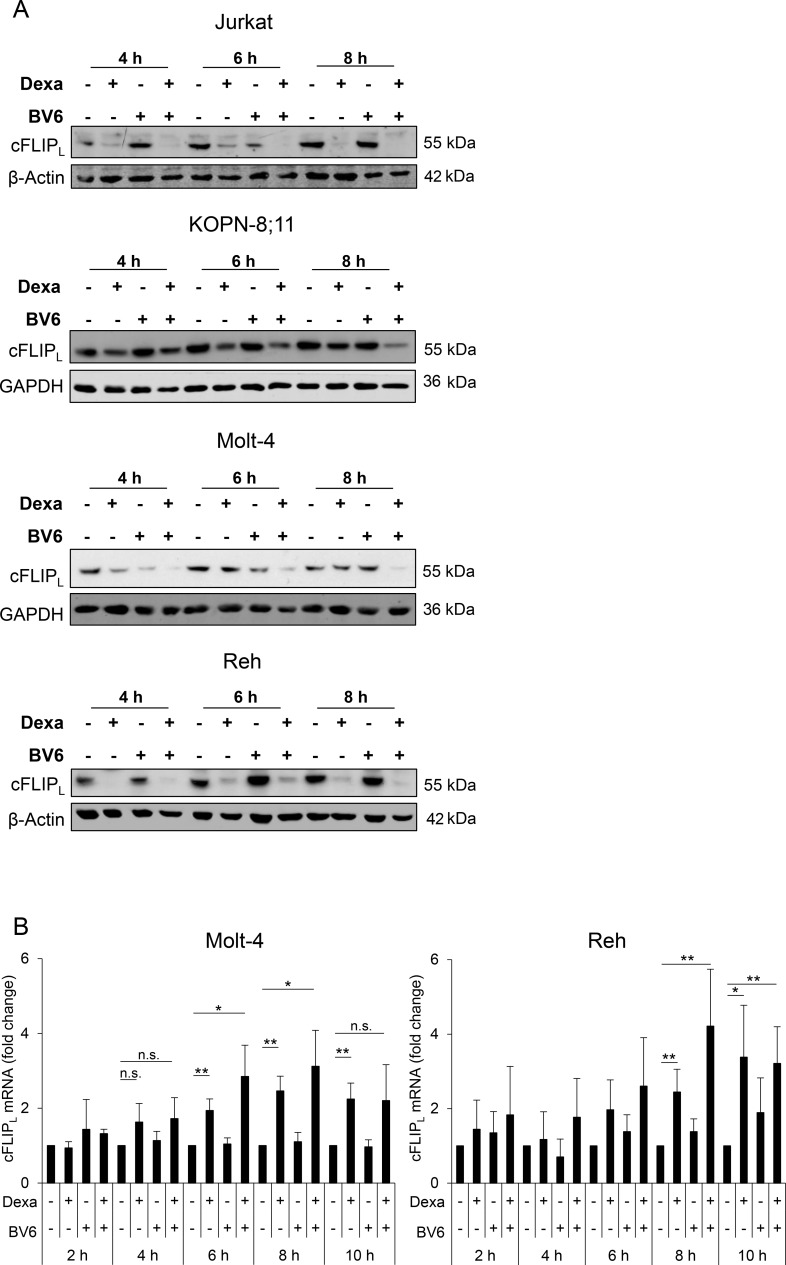
Dexa downregulates cFLIP_L_ protein in ALL cells ALL cells were treated for indicated time points with Dexa and/or BV6 (Jurkat: 300 µM Dexa, 7 µM BV6; KOPN-8;11: 150 µM Dexa, 2 µM BV6; Molt-4: 300 µM Dexa, 5 µM BV6; Reh: 300 µM Dexa, 0.3 µM BV6). (**A**) Protein expression of cFLIP_L_ was analyzed by Western blotting. β-Actin or GAPDH served as loading control. (**B**) cFLIP_L_ mRNA levels were analyzed using qRT-PCR. Fold change of cFLIP_L_ mRNA as mean and standard deviation (SD) of at least three independent experiments are shown.

Initially, we determined protein expression of the two major isoforms of cFLIP (i.e. cFLIP_L_ and cFLIP_S_) in ALL cell lines. Since all analyzed ALL cell lines predominately expressed cFLIP_L_ rather than cFLIP_S_ protein (Supplementary Figure 1), we focused our analysis in ALL on cFLIP_L_. We then asked whether Dexa as single agent or in combination with BV6 affects cFLIP_L_ levels. Interestingly, treatment with Dexa alone or together with BV6 downregulated cFLIP_L_ protein levels already after a few hours (Figure 1A). To examine whether the loss of cFLIP_L_ protein is due to changes in mRNA expression, we performed qRT-PCR analysis. Dexa treatment increased rather than suppressed cFLIP_L_ mRNA levels (Figure 1B), indicating that the observed loss of cFLIP_L_ protein by Dexa or Dexa/BV6 treatment is independent of mRNA expression. These data indicate a Dexa-mediated downregulation of cFLIP_L_ protein independent of mRNA expression.

### Dexa impedes BV6-stimulated NF-κB activation

**Figure 2:**
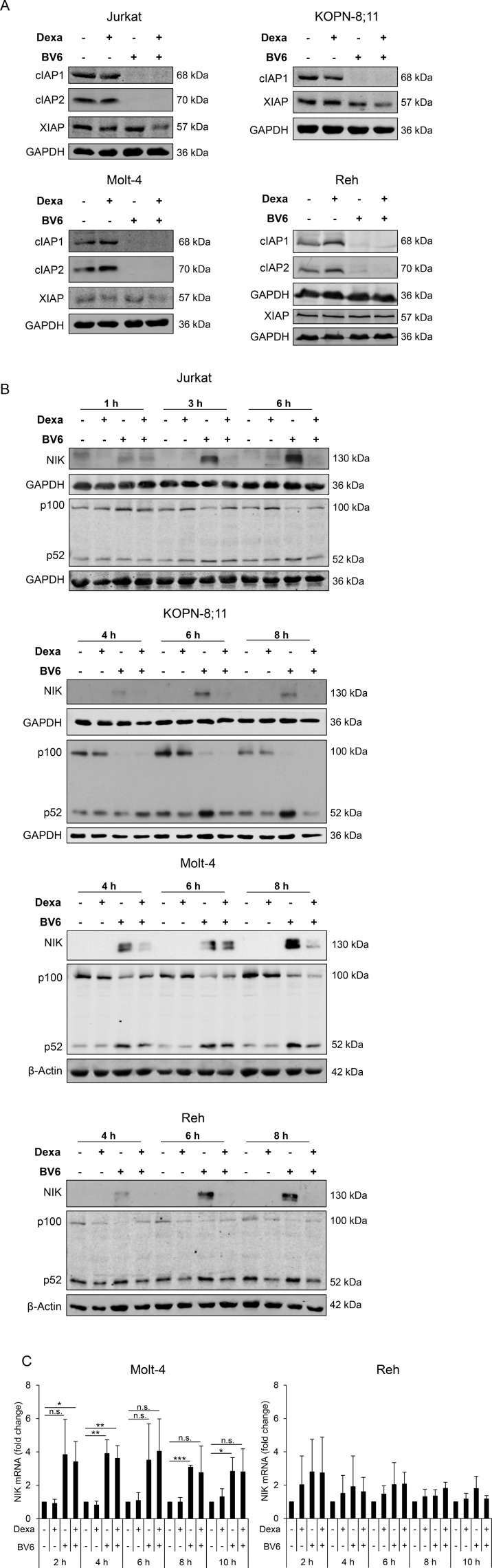
Dexa impedes BV6-stimulated NF-κB activation ALL cells were treated for four hours (**A**) or indicated time points (**B**, **C**) with Dexa and/or BV6 (Jurkat: 300 µM Dexa, 7 µM BV6; KOPN-8;11: 150 µM Dexa, 2 µM BV6; Molt-4: 300 µM Dexa, 5 µM BV6; Reh: 300 µM Dexa, 0.3 µM BV6). (**A**) Protein expression of cIAP1, cIAP2 and XIAP was analyzed by Western blotting. GAPDH served as loading control. (**B**) Protein expression of NIK and p100/p52 was analyzed by Western blotting. β-Actin or GAPDH served as loading control. (**C**) NIK mRNA levels were analyzed using qRT-PCR. Fold change of NIK mRNA as mean and SD values of at least three independent experiments are shown.

Since Smac mimetics have been described to deplete IAP proteins [16, 17], we determined expression levels of cellular IAP (cIAP)1, cIAP2 and x-linked IAP (XIAP) upon treatment with Dexa and BV6. As expected, treatment with either BV6 alone or in combination with Dexa caused a loss of cIAP1 and cIAP2 in all four cell lines and XIAP expression slightly decreased by Dexa/BV6 cotreatment (Figure 2A). In KOPN-8;11 cells, we did not detect cIAP2 protein (Figure 2A).

As loss of cIAP proteins can lead to activation of the non-canonical NF-κB pathway [16, 17], we next assessed expression of NF-κB-inducing kinase (NIK) and p100/p52 as key components of non-canonical NF-κB signaling. Treatment with BV6 caused accumulation of NIK protein (Figure 2B), in line with the observed BV6-imposed depletion of cIAP proteins (Figure 2A), which serve as E3 ligases of NIK [16]. In addition, BV6 increased NIK mRNA expression (Figure 2C). Interestingly, addition of Dexa abolished the BV6-mediated accumulation of NIK protein (Jurkat, KOPN-8;11, Reh) or reduced it (Molt-4) (Figure 2B). Consistently, addition of Dexa slightly diminished the BV6-mediated processing of the NF-κB precursor p100 to p52 in all four cell lines (Figure 2B). Altogether these data indicate that Dexa impedes the BV6-triggered activation of NF-κB.

### Dexa-stimulated downregulation of cFLIP_L_ protein occurs largely independent of the proteasome, lysosomal enzymes and caspases

**Figure 3:**
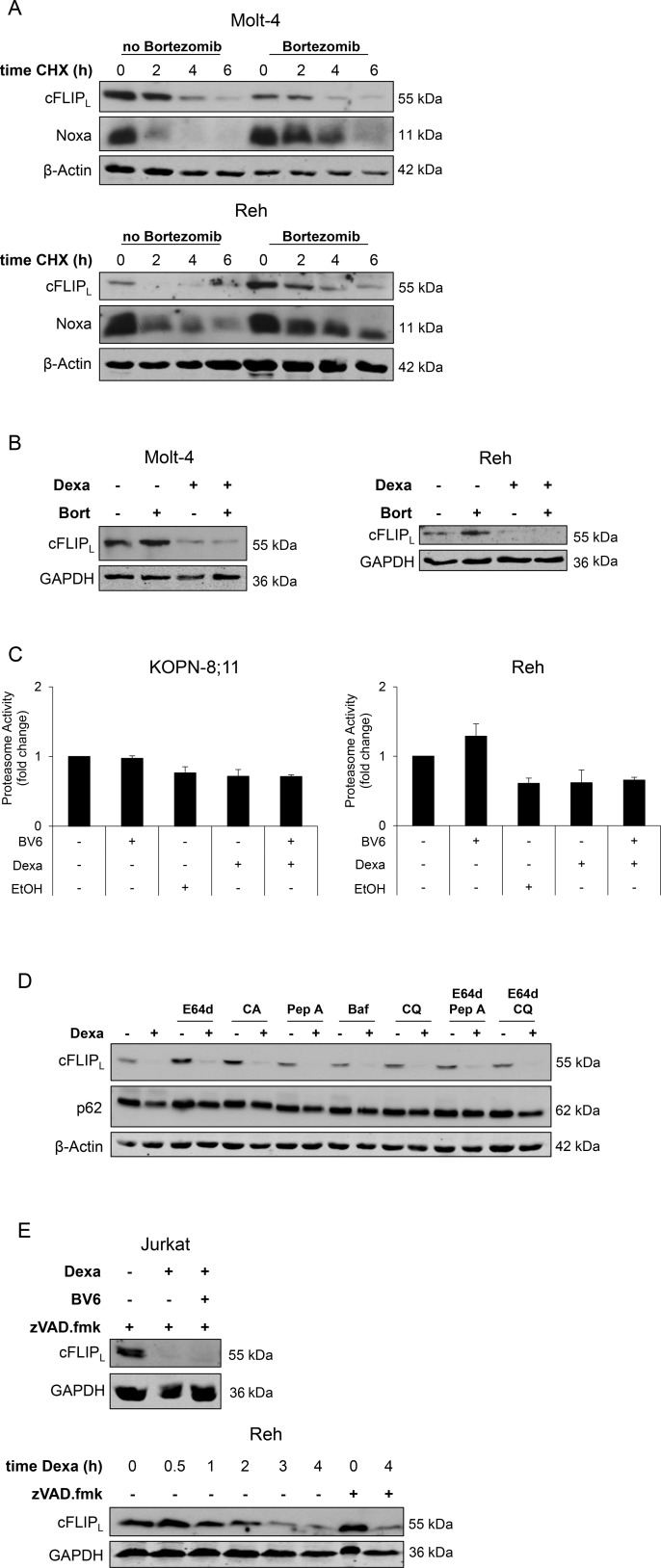
Dexa-stimulated downregulation of cFLIP_L_ protein occurs largely independent of the proteasome, lysosomal enzymes and caspases (**A**) Molt-4 and Reh cells were treated with 30 nM Bortezomib to block proteasomal activity, after one hour cells were treated with 10 ng/ml CHX for indicated time points. Expression of cFLIP_L_ and Noxa was analyzed by Western blotting. β-Actin served as loading control. (**B**) Molt-4 and Reh cells were treated with 30 nM Bortezomib (Bort) to block proteasomal activity, after one hour cells were treated with 300 µM Dexa for four hours. Expression of cFLIP_L_ was analyzed by Western blotting. GAPDH served as loading control. (**C**) Proteasome activity was analyzed using Chemicon 20S Proteasome Activity Assay. Fold change of proteasome activity of two independent experiments performed in duplicates is shown. EtOH was used as solvent for Dexa. (**D**) Reh cells were incubated with inhibitors of lysosomal enzymes (10 µg/ml E64d, 10 µg/ml CA-074 methyl ester (CA), 10 µg/ml Pepstatin A (Pep A), 50 nM Bafilomycin A (Baf), 25 µM Chloroquine (CQ)) for one hour, followed by treatment with 300 µM Dexa for four hours. cFLIP_L_ protein expression was analyzed using Western blotting. β-Actin served as loading control. (**E**) Jurkat and Reh cells were treated with 20 µM zVAD.fmk and 300 µM Dexa for four hours or indicated time points. cFLIP_L_ expression was analyzed by Western blotting. GAPDH served as loading control.

Since our data point to a transcription-independent regulation of cFLIP_L_, we investigated whether Dexa-stimulated downregulation of cFLIP_L_ is due to changes in cellular protein degradation pathways. cFLIP_L_ is described as short-lived protein, which is primarily regulated by the ubiquitin-proteasomal pathway [18]. To determine the half-life of cFLIP_L_ in ALL cell lines, we performed cycloheximide (CHX) chase assays to assess cFLIP_L_ levels upon inhibition of protein synthesis by CHX. CHX treatment caused a rapid decrease in cFLIP_L_ protein (Figure 3A). To analyze the role of the proteasomal pathway, we blocked the proteasome by the specific inhibitor Bortezomib. The addition of Bortezomib delayed loss of cFLIP_L_ protein upon protein synthesis inhibition, in particular in Reh cells, but did not completely rescue it, whereas loss of Noxa protein, a known target of the proteasome, was partially restored in both cell lines (Figure 3A), indicating that cFLIP_L_ is not strictly regulated by the proteasome in the analyzed cell lines. To explore whether loss of cFLIP_L_ protein upon Dexa treatment is mediated via the proteasome, we added Bortezomib to Dexa-treated cells. Interestingly, Bortezomib failed to rescue Dexa-mediated loss of cFLIP_L_ protein (Figure 3B). To assess whether Dexa directly impairs proteasome activity, we performed a proteasome activity assay. Dexa alone or in combination with BV6 did not alter 20S proteasome activity (Figure 3C). By comparison, Dexa treatment had little effects on other short-lived proteins at early time points, such as Noxa or Mcl-1 (Supplementary Figure 2).

To investigate whether cFLIP_L_ protein is degraded upon Dexa treatment via the lysosomal pathway, we blocked lysosomal enzymes by different pharmacological inhibitors. All of them (alone or in combination) failed to prevent loss of cFLIP_L_ protein after Dexa treatment (Figure 3D). Since cFLIP_L_ is a known target of caspase-8, we analyzed whether caspase-mediated cleavage of cFLIP_L_ is responsible for its loss upon Dexa treatment. But the addition of N-benzyloxycarbonyl-Val-Ala-Asp-(OMe)-fluoromethylketone (zVAD.fmk), a pan-caspase inhibitor, did not prevent Dexa-mediated downregulation of cFLIP_L_ (Figure 3E). This set of experiments indicates that Dexa-mediated loss of cFLIP_L_ protein in ALL cell lines is not primarily mediated via the proteasome, lysosomal enzymes or caspases.

### High cFLIP_L_ levels impair Dexa/BV6-mediated cell death in Reh cells

**Figure 4:**
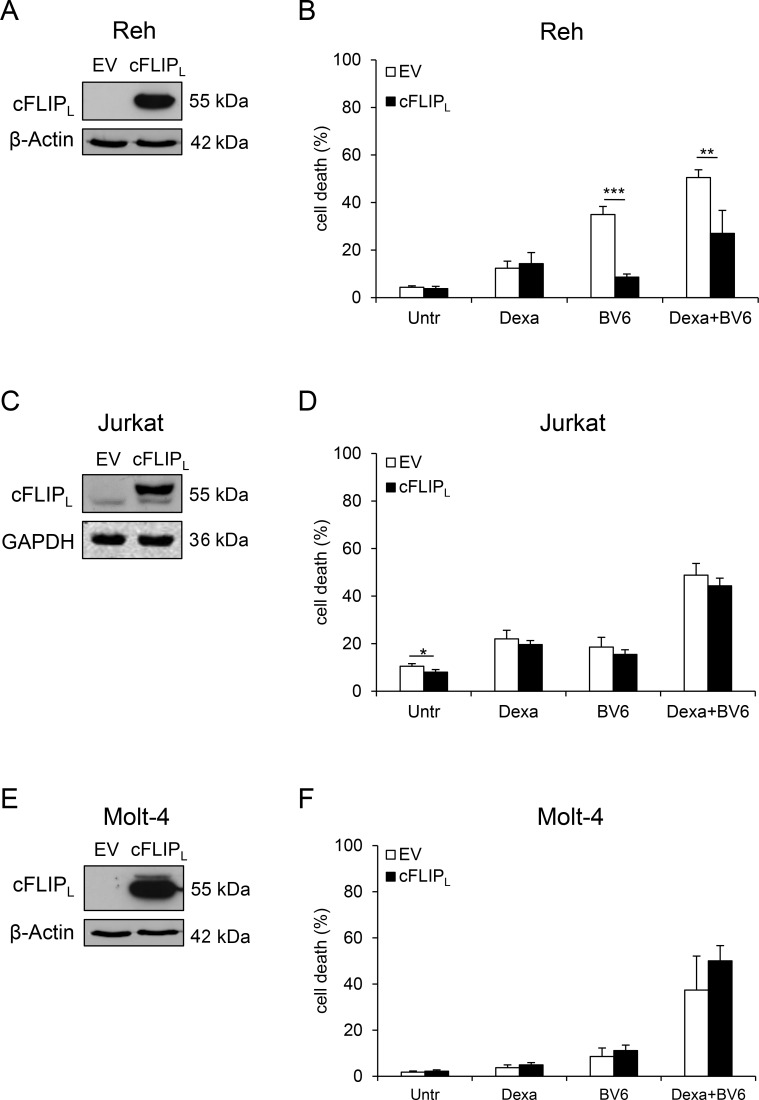

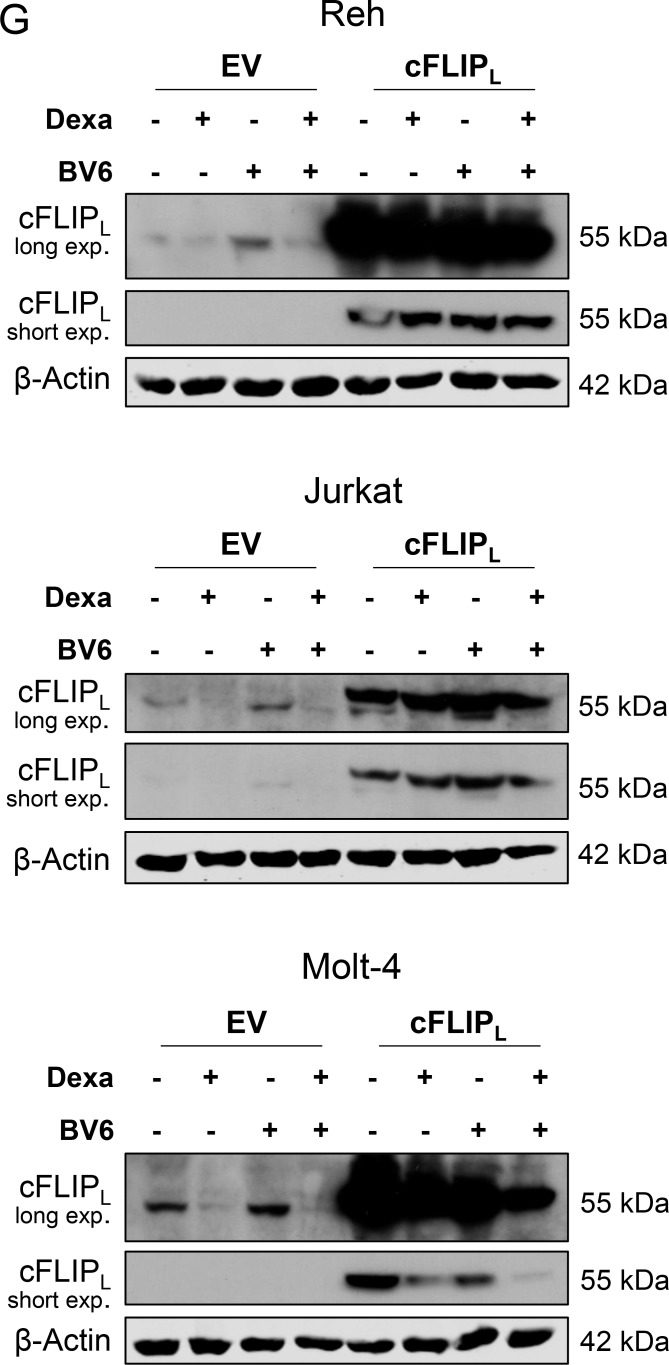

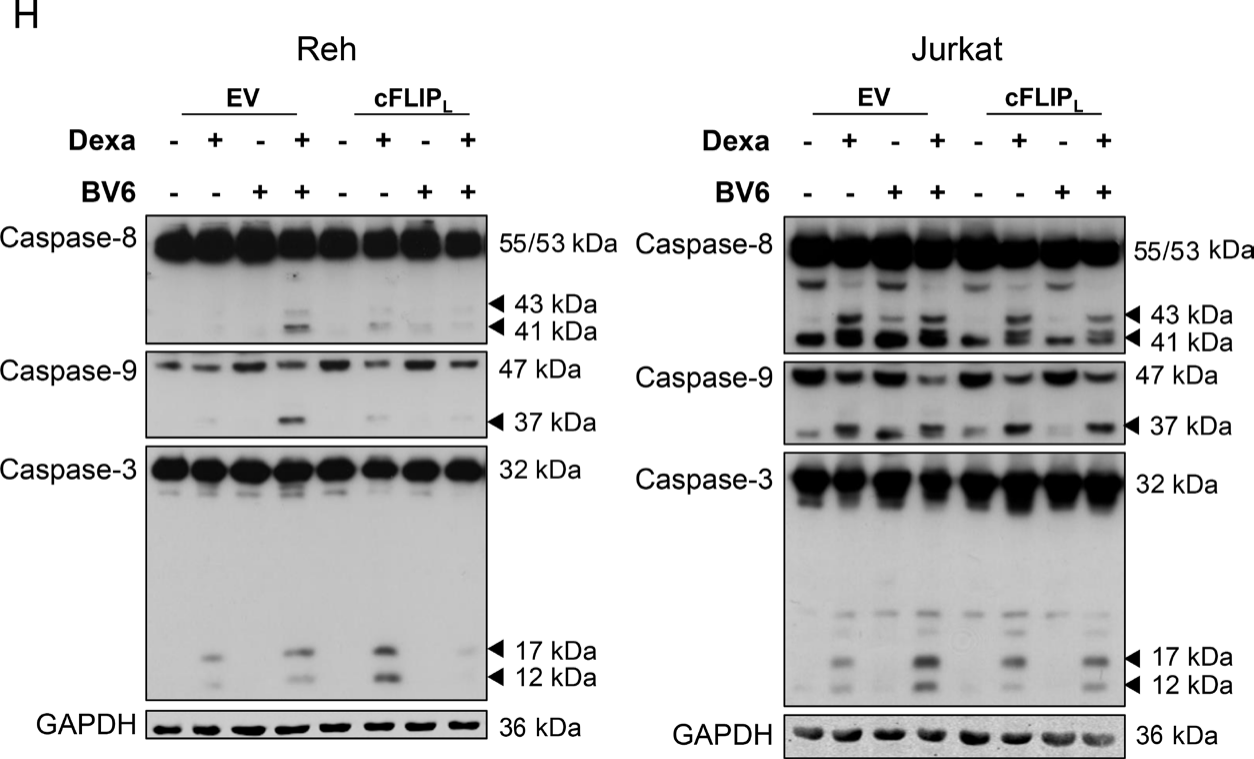
High cFLIP_L_ levels impair Dexa/BV6-mediated cell death (**A**, **C**, **E**) Protein levels of cFLIP_L_ were assessed by Western blotting in EV or cFLIP_L_ overexpressing (cFLIP_L_) cells. GAPDH served as loading control. (**B**, **D**, **F**) cFLIP_L_ overexpressing (OE) cells were treated with Dexa and/or BV6 (Jurkat: 300 µM Dexa; 7 µM BV6; Molt-4: 300 µM Dexa, 5 µM BV6; Reh: 300 µM Dexa, 0.3 µM BV6) for 24 hours (Reh, Molt-4) or 15 hours (Jurkat), respectively, and cell death was determined by FSC/SSC analysis and flow cytometry. Mean and SD of at least three independent experiments performed in triplicate are shown; ^**^p < 0.01, ^***^p < 0.001. (**G**) OE cells were treated with 300 µM Dexa and/or BV6 (Jurkat: 7 µM BV6; Molt-4: 5 µM BV6; Reh: 0.3 µM BV6) for four hours. cFLIP_L_ expression was assessed by Western blotting. β-Actin served as loading control. (**H**) Reh and Jurkat OE cells were treated with 300 µM Dexa and/or BV6 (Jurkat: 7 µM BV6; Reh: 0.3 µM BV6) for six hours. Pro-caspase expression and caspase cleavage were analyzed by Western blotting. GAPDH served as loading control.

To explore the functional relevance of cFLIP_L_ in Dexa/BV6-mediated cell death, we created cell lines stably overexpressing cFLIP_L_ (Figure 4A, 4C, 4E). Of note, cFLIP_L_ overexpression significantly reduced Dexa/BV6- as well as BV6-mediated cell death in Reh cells (Figure 4B), in which the ectopically expressed cFLIP_L_ protein was resistant to Dexa/BV6-imposed downregulation (Figure 4G). In Jurkat and Molt-4 cells, Dexa/BV6 downregulated ectopically expressed cFLIP_L_ in addition to endogenous cFLIP_L_ protein (Figure 4G), consistent with the failure of cFLIP_L_ overexpression to rescue Dexa/BV6-induced cell death in these cell lines (Figure 4D, 4F). Control experiments confirmed that cFLIP_L_ overexpression significantly reduced TNFα/BV6-mediated cell death in Jurkat and Molt-4 cells (Supplementary Figure 3). In line with the cFLIP_L_-conferred protection from Dexa/BV6-induced cell death (Figure 4B), cFLIP_L_ overexpression reduced Dexa/BV6-imposed cleavage of caspase-8, caspase-9 and caspase-3 compared to Reh control cells expressing empty vector (EV) (Figure 4H), whereas overexpression of cFLIP_L_ had little effects on Dexa/BV6-induced caspase cleavage in Jurkat cells (Figure 4H). These data show that high cFLIP_L_ levels impair Dexa/BV6-mediated cell death in a cell line-dependent manner.

### Knockdown of cFLIP increases BV6-mediated cell death

**Figure 5:**
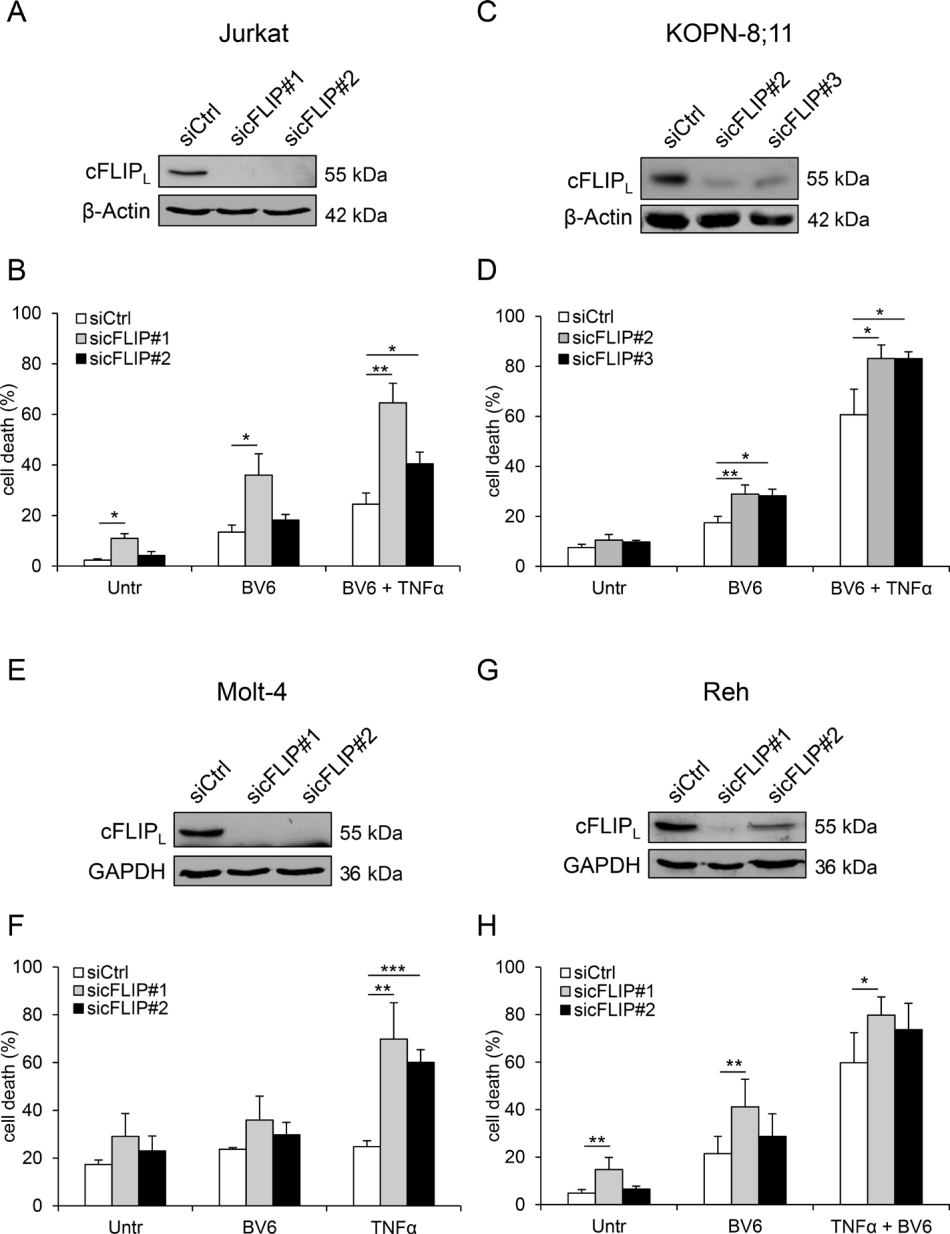
Knockdown of cFLIP increases BV6-mediated cell death Cells were transiently transfected with two distinct siRNAs targeting cFLIP or control siRNA. (**A**, **C**, **E**, **G**) Protein expression of cFLIP_L_ was analyzed by Western blotting. β-Actin or GAPDH served as loading control. (**B**, **D**, **F**, **H**) Cells were treated with BV6 and/or TNFα (Jurkat: 7 µM BV6, 1 ng/ml TNFα; KOPN-8;11 µM BV6, 1 ng/ml TNFα; Molt-4: 5 µM BV6, 100 ng/ml TNFα; Reh: 0.1 µM BV6, 0.05 ng/ml TNFα) for 24 hours or 15 hours (Jurkat), respectively. Cell death was determined by FSC/SSC analysis and flow cytometry. Mean and SD of at least three independent experiments performed in triplicate are shown; ^*^p < 0.05, ^**^p < 0.01, ^***^p < 0.001.

Next, we tested the functional relevance of Dexa-mediated loss of cFLIP protein to sensitize ALL cells to BV6 by siRNA-mediated knockdown of cFLIP to mimic its depletion by Dexa. In all tested cell lines, knockdown of cFLIP by using two independent siRNA sequences (Figure 5A, 5C, 5E, 5G) caused an increase in TNFα/BV6-mediated cell death that served as positive control (Figure 5B, 5D, 5F, 5H). Importantly, cFLIP knockdown significantly increased BV6-mediated cell death in KOPN-8;11 cells (Figure 5D). In Jurkat and Reh cells, cFLIP knockdown by using sequence #1 significantly enhanced cell death in BV6-treated cells (Figure 5B, 5G), while cFLIP silencing did not alter BV6-induced cell death in Molt-4 cells (Figure 5F). These results demonstrate that cFLIP silencing increases BV6-mediated cell death in a cell line-dependent manner.

## DISCUSSION

### Regulation of Smac mimetic-induced cell death by cFLIP

In the present study, we discover that Dexa-imposed suppression of cFLIP_L_ contributes to Dexa/BV6-induced cell death. The conclusion that cFLIP_L_ negatively regulates Dexa/BV6-induced cell death is supported by our data showing that overexpression of cFLIP_L_ to an extent that is no longer subject to Dexa-mediated downregulation rescues Dexa/BV6-mediated cell death. In addition, knockdown of cFLIP mimics Dexa treatment in ALL, as it increases BV6-mediated cell death. All these findings underscore the functional relevance of cFLIP in Dexa/BV6-mediated cell death. Since we previously demonstrated that Dexa/BV6-induced depletion of cIAP proteins is followed by ripoptosome formation [15], which is known to be negatively regulated by cFLIP [10, 11, 19], reduction of cFLIP protein levels by Dexa/BV6 treatment may well promote ripoptosome formation and thereby cell death. Thus, in addition to downregulating cIAP proteins, Dexa/BV6-induced suppression of cFLIP contributes to Dexa/BV6-mediated cell death.

cFLIP is a well-described negative regulator of cell death in many tumor entities and treatment strategies. cFLIP has been shown to protect from Smac mimetic-induced cell death [20, 21] and silencing of cFLIP was found to sensitize different tumor cell lines to Smac mimetics [21, 22]. Also, there are several reports showing that cFLIP can protect cancer cells from TRAIL-, CD95- or chemotherapy-induced cell death [23–26].

### Molecular mechanisms of cFLIP downregulation

Furthermore, our study provides new insights into the molecular mechanisms that are responsible for Dexa-imposed downregulation of cFLIP protein. cFLIP expression is tightly regulated by various transcriptional and post-transcriptional mechanisms. While cFLIP is known to be regulated by different transcription factors including NF-κB [27, 28], our data point to a transcription-independent regulation of cFLIP by Dexa, as Dexa suppressed cFLIP_L_ protein but not mRNA levels. Nevertheless, Dexa attenuated BV6-stimulated non-canonical NF-κB activation in ALL cell lines, which is consistent with other reports showing that glucocorticoids such as Dexa can block NF-κB [29–31].

cFLIP is known as a short-lived protein and its turnover has been shown to determine sensitivity to cell death, e.g. to death receptor signals [32]. Several E3 ubiquitin ligases, for example Itch [33, 34], have been identified that polyubiquitinate cFLIP to induce its proteasome-mediated proteolysis, and proteasome inhibitors have been described to rescue the degradation of cFLIP protein [35, 36]. In addition, increased proteasome activity is associated with downregulation of cFLIP protein [37]. However, our findings suggest that Dexa-induced loss of cFLIP_L_ protein is not primarily due to increased proteasomal degradation, since i) addition of the proteasome inhibitor Bortezomib failed to fully protect from Dexa-induced loss of cFLIP protein and since ii) treatment with Dexa alone or in combination with BV6 did not alter 20S proteasomal activity.

Besides proteasome-mediated proteolysis, tumor necrosis factor (TNF) receptor-associated factor (TRAF)7, another E3 ubiquitin ligase, has been described to polyubiquitinate cFLIP and to induce its lysosomal degradation [38]. However, our findings suggest a lysosome-independent downregulation of cFLIP_L_, as inhibition of several lysosomal enzymes, either alone or in combination, failed to rescue Dexa-stimulated cFLIP_L_ degradation. It is also unlikely that caspase-8-triggered cleavage of cFLIP_L_ is responsible for its downregulation by Dexa, since the pan-caspase inhibitor zVAD.fmk did not prevent Dexa-mediated loss of cFLIP_L_.

As transcriptional or post-translational regulation, e.g. by caspases or the proteasome, are not primarily responsible for downregulation of cFLIP_L_, it might be that translational processes are affected by Dexa. While in ALL cells glucocorticoids have been shown to repress genes involved in RNA, protein and nucleotide synthesis [39], which is in line with studies on other tumor entities [40] or tissues [41, 42], Dexa has recently been reported to not alter mRNA translation in ALL cell lines [43]. While the rapid kinetic of Dexa-mediated loss of cFLIP_L_ protein similar to inhibition of protein synthesis by CHX is consistent with a block of translation or ribosomal proteins upon Dexa treatment, further studies are required to fully understand the mechanisms underlying Dexa-stimulated decrease of cFLIP_L_.

By showing that Dexa-mediated downregulation of cFLIP_L_ contributes to its sensitization to BV6-induced cell death, our study provides new insights into the molecular mechanisms of the cooperative induction of cell death by Dexa/BV6. As glucocorticoids are part of treatment regimens for ALL patients, our findings showing that Dexa and BV6 cooperate to induce cell death provide new concepts to enhance glucocorticoid sensitivity.

## MATERIALS AND METHODS

### Cell culture and chemicals

ALL cell lines were obtained from DSMZ (Braunschweig, Germany). Cells were cultured in RPMI 1640 medium (Life Technologies/Thermo Fisher Scientific, Darmstadt, Germany). Media were supplemented with 10% FCS (fetal calf serum) (Life Technologies/Thermo Fisher Scientific), 1 mM pyruvate (Life Technologies/Thermo Fisher Scientific), 25 mM HEPES (Life Technologies/Thermo Fisher Scientific) and 1% penicillin/streptomycin (Life Technologies/Thermo Fisher Scientific). The Smac mimetic BV6 was kindly provided by Genentech, Inc. (South San Francisco, CA, USA). The glucocorticoid Dexa was purchased from Sigma-Aldrich (Steinheim, Germany), the caspase inhibitor zVAD.fmk from Bachem (Heidelberg, Germany), the proteasome inhibitor Bortezomib from Selleckchem (Houston, TX, USA) and cycloheximide (CHX), E64d, CA-074 methyl ester, chloroquine, pepstatin A and Bafilomycin A from Sigma-Aldrich. All other chemicals were purchased from Sigma-Aldrich (Steinheim, Germany) or Carl Roth (Karlsruhe, Germany) unless indicated otherwise.

### Determination of cell death and proteasome activity

Cell death was assessed by forward/side scatter (FSC/SSC) analysis and flow cytometry (FACS Canto II; BD Biosciences). 20S Proteasome activity was analyzed using CHEMICON 20S Proteasome Activity Assay Kit according to the manufacturer’s instructions (Merck, Darmstadt, Germany).

### Western blot analysis

Western blot analysis was performed as described previously [44] using the following antibodies: Mouse anti-cFLIP (Enzo Life Sciences, Lörrach, Germany), goat anti-cIAP1 (R&D Systems, Wiesbaden, Germany), rat anti-cIAP2 (Enzo Life Sciences), mouse anti-XIAP (BD Biosciences, Heidelberg, Germany), rabbit anti-NIK (Cell Signaling Technologies, Beverly, MA USA), mouse anti-p100/p52 (Merck Millipore, Darmstadt, Germany), rabbit anti-p62 (MBL International, Woburn, MA, USA), mouse anti-Noxa (Enzo Life Sciences), rabbit anti-Mcl-1 (Enzo Life Sciences), mouse anti-caspase-8 (Enzo Life Sciences), rabbit anti-caspase-3 (Cell Signaling Technologies), rabbit anti-caspase-9 (Cell Signaling Technologies), mouse anti-β-actin (Sigma-Aldrich), mouse anti-GAPDH (HyTest, Turku, Finland), mouse anti-tubulin (Calbiochem, Merck Millipore, Darmstadt, Germany). Secondary antibodies conjugated to horseradish peroxidase (Santa Cruz Biotechnology, Santa Cruz, CA, USA) were used for enhanced chemiluminescence detection (Amersham Bioscience, Freiburg, Germany). Alternatively, secondary antibodies labeled with IRDye infrared dyes were used for fluorescence detection (Odyssey Imaging System, LI-COR Bioscience, Bad Homburg, Germany). All Western blots shown are representative of two or three independent experiments.

### qRT-PCR analysis

Total RNA was isolated using peqGOLD Total RNA Kit (peqlab/VWR, Darmstadt, Germany), cDNA synthesis was performed with 1 µg RNA using RevertAid H Minus First Strand cDNA Synthesis Kit (Molecular Biology/Thermo Fisher Scientific). To quantify gene expression qRT-PCR analysis was performed using SYBR Green Reaction Mix (Applied Biosystems, Darmstadt, Germany) with 7900HT Fast Real-Time PCR System (Applied Biosystems). The following primers were used: 28S (forward: 5′-TTGAAAATCCGGGGGAGAG-3′; reverse: 5′-ACATTGTTCCAACATGCCAG-3′), 18S (forward: 5′-C GCAAATTACCCACTCCCG-3′; reverse: 5′-TTCCAAT TACAGGGCCTCGAA-3′), glucose 6-phosphate dehydrogenase (G6PDH) (forward: 5′-ATCGACCAC TACCTGGGCAA-3′; reverse: 5′-AGGCCCTGCACTACG TCTT-3′), RNA polymerase II (forward: 5′-GCACCACG TCCAATGACAT-3′; reverse: 5′-AATACCTTCGTCGG CGTG-3′), cFLIP (forward: 5′-GCTCACCATCCCTGTA CCTG-3′; reverse: 5′-CAGGAGTGGGCGTTTTCTT-3′), NIK (forward: 5′- CCAGCTGCCATCTCTATCATC-3′; reverse: 5′-AAAAAGTGGGGCTGAACTCT-3′). mRNA expression levels of genes of interest were normalized to three housekeeping genes and relative expression of target gene transcript and reference gene transcripts was calculated as ΔΔCt.

### Gene silencing and transduction

Knockdown experiments with small interfering RNA (siRNA) were performed using Neon Transfection System (Invitrogen, Karlsruhe, Germany) according to the manufacturer’s instructions [45], using 80 nM Silencer^®^Select siRNAs (Thermo Fisher Scientific) against cFLIP (#1: s16864, #2: s16865, #3: s229912), or non-targeting control siRNA (no. 4390843). Jurkat, KOPN-8;11 and Molt-4 cells were treated 48 hours after transfection. Reh cells were transfected twice at intervals of 48 hours and treated 24 hours after the second transfection. Overexpression of cFLIP was performed by retroviral transduction using cFLIP_L_ and the pBABE-puro retroviral vector system. For virus production, Phoenix cells were transfected with 20 µg DNA using calcium phosphate method. Virus-containing supernatant of Phoenix cells was filtered and added via spin transduction in the presence of 4 µg/ml protamine sulfate (Sigma-Aldrich) to Jurkat, Molt-4 or Reh cells. Transduced cells were selected with 5 µg/ml puromycin (Sigma-Aldrich).

### Statistical analysis

Statistical significance was assessed using Student’s *t*-Test (two-tailed distribution, equal variance) calculated with Microsoft Excel (Munich, Germany).

## SUPPLEMENTARY MATERIALS FIGURES


