# Supplementary material for: Regulation of the antiapoptotic protein cFLIP by the glucocorticoid Dexamethasone in ALL cells
Source: Oncotarget. 2018 Mar 27;9(23):16521–32. doi: 10.18632/oncotarget.24782 (PMC5893259; doi:10.18632/oncotarget.24782)
Supplement: Supplementary file 1 [file oncotarget-09-16521-s001.pdf]

# Regulation of the antiapoptotic protein cFLIP by the glucocorticoid Dexamethasone in ALL cells

## SUPPLEMENTARY MATERIALS

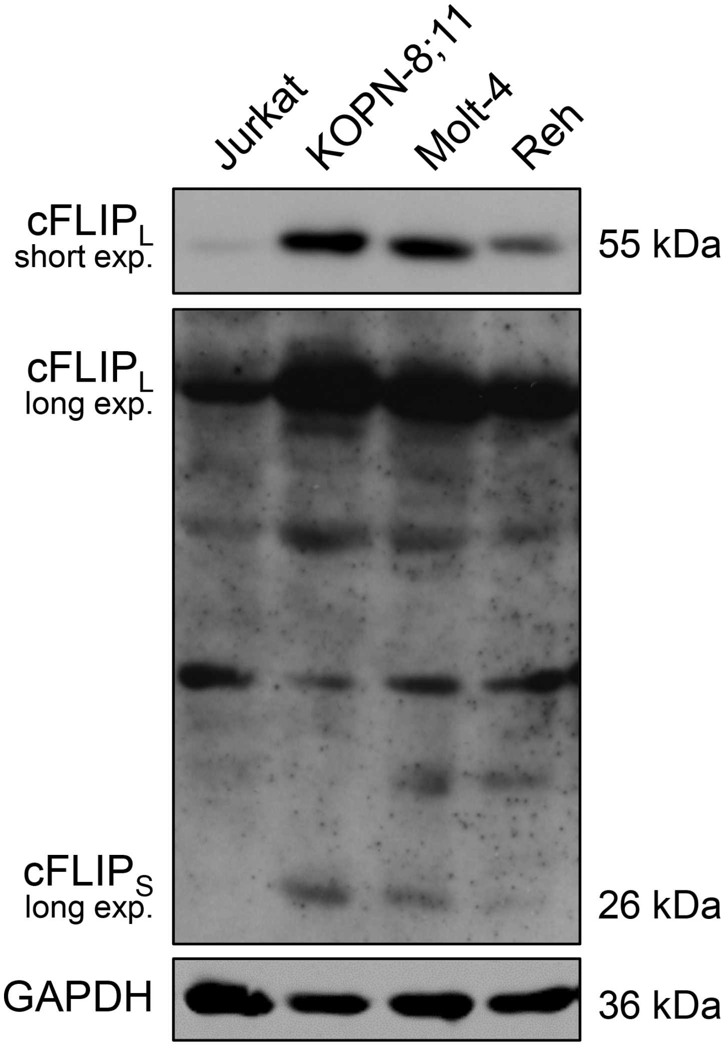


**Supplementary Figure 1: cFLIP expression in ALL cell lines.** Protein expression of $cFLIP_L$ was analyzed by Western blotting. GAPDH served as loading control.

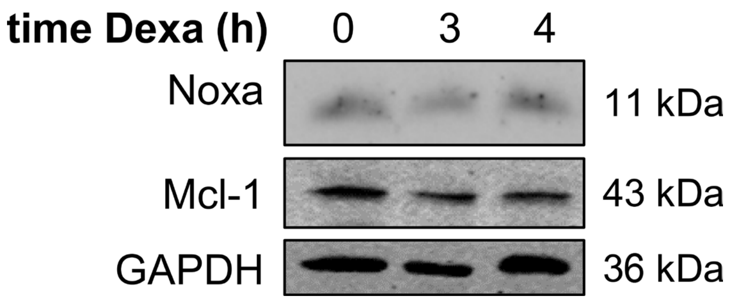


**Supplementary Figure 2: Noxa and Mcl-1 expression upon Dexa treatment.** Reh cells were treated with 300 μM Dexa for indicated time points. Noxa and Mcl-1 expression was analyzed by Western blotting. GAPDH served as loading control..

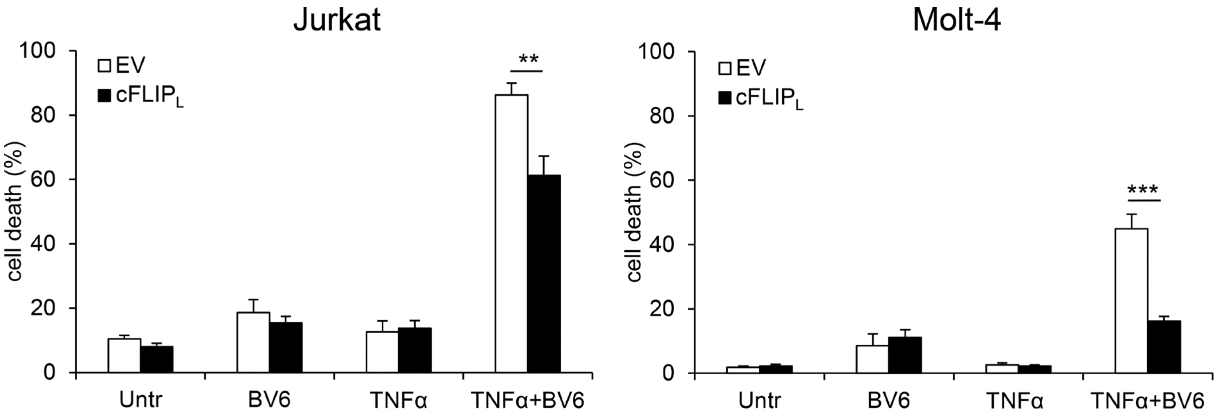


**Supplementary Figure 3: TNFα/BV6-induced cell death in cFLIP OE cells.** cFLIPL overexpressing cells were treated with BV6 and/or TNFα (Jurkat: 7 μM BV6, 1 ng/ml TNFα; Molt-4: 5 μM BV6, 100 ng/ml TNFα) for 15 hours (Jurkat) or 24 hours (Molt-4). Cell death was determined by FSC/SSC analysis and flow cytometry. Mean and SD of at least three independent experiments performed in triplicate are shown; $^{*}p < 0.05$, $^{**}p < 0.01$, $^{***}p < 0.001$.
